# Insomnia in medical students during an examination period: associations with depression, dysfunctional sleep beliefs, and academic performance

**DOI:** 10.1186/s12909-026-10565-4

**Published:** 2026-10-12

**Authors:** Maximilian Bailer, Kardo Almazury, Franziska Sonnauer, Maximilian I. Sprügel, Stefan Mestermann, Janine Utz, Johannes Kornhuber, Philipp Spitzer

**Affiliations:** 1 Department of Psychiatry and Psychotherapy, University Hospital Erlangen, Friedrich-Alexander-Universität Erlangen-Nürnberg, Schwabachanlage 6, Erlangen, 91054 Germany; 2 Department of Neurology, University Hospital Erlangen, Friedrich- Alexander-Universität Erlangen-Nürnberg, Erlangen, Germany; 3 Department of Child and Adolescent Mental Health, University Hospital Erlangen, Friedrich-Alexander-Universität Erlangen-Nürnberg, Erlangen, Germany

**Keywords:** Insomnia Severity Index (ISI), Dysfunctional beliefs about sleep, Medical students, Sleep disorders, Insomnia, Chronotype, Academic performance, Academic stress, Depressive symptoms

## Abstract

**Background:**

Insomnia is common among medical students and is linked to impaired mental health and academic functioning. Medical students are exposed to persistent psychosocial stress, yet the extent to which state factors (e.g., stress, mood) versus trait factors (e.g., chronotype, sleep beliefs) contribute to insomnia severity is unclear. While dysfunctional sleep beliefs are relatively stable cognitive traits, they remain amenable to targeted interventions.

**Methods:**

We conducted a cross-sectional survey at a German medical school during a fourth year exam period. Students completed the Insomnia Severity Index (ISI; primary outcome), selected items of the Pittsburgh Sleep Quality Index (PSQI), the Patient Health Questionnaire-9 (PHQ-9), the Generalized Anxiety Disorder-2 (GAD-2), a visual analogue scale (VAS) for stress, the Dysfunctional Beliefs and Attitudes about Sleep Scale-16 (DBAS-16), and reduced the Morningness–Eveningness Questionnaire (rMEQ); academic performance was recorded. Group differences were tested with t tests and chi-square tests. A multivariable linear regression modeled insomnia severity (ISI) as a function of state and trait variables; a secondary model examined DBAS-16 subscales.

**Results:**

Of 124 students, 46.0% (57/124) screened positive for clinically relevant insomnia symptoms (ISI > 10). Students with clinically relevant inomnias symptoms had shorter total sleep time (391.0 ± 76.5 vs. 438.2 ± 58.3 min; *P* < 0.001), longer sleep onset latency (51.1 ± 37.1 vs. 21.6 ± 18.0 min; *P* < 0.001), and lower sleep efficiency (82.2% ± 12.2% vs. 90.1% ± 7.2%; *P* < 0.001). In adjusted analyses (R² = 0.572; adjusted R² = 0.523), higher PHQ-9 (β = 0.30; *P* = 0.01) and DBAS-16 (β = 0.28; *P* < 0.001) were independently associated with higher ISI, whereas higher sleep efficiency (β = −0.30; *P* < 0.001) and academic performance (β = −0.15; *P* = 0.037) were inversely associated; perceived stress, anxiety, and chronotype were not independent correlates. In the DBAS-16 subscale model (R² = 0.332; adjusted R² = 0.307), Worry/Helplessness and Perceived Consequences were significantly associated with higher ISI.

**Conclusions:**

Clinically relevant insomnia symptoms were frequent among medical students. Depressive symptoms and dysfunctional sleep beliefs were key correlates of insomnia severity, and academic performance was inversely related. These findings suggest that sleep health and mental well-being deserve attention in medical education and student health services.

**Supplementary Information:**

The online version contains supplementary material available at https://doi.org/10.1186/s12909-026-10565-4.

## Background

Medical students frequently exhibit symptoms of stress and exhaustion, as well as stress-related illnesses such as depression [1–3]. Concurrently, intrinsic academic motivation often declines among medical students over the course of their university education [4], which may be related to psychosocial stressors encountered during medical training, including impaired sleep quality [5]. 

Sleep disorders, especially insomnia, are closely associated with stress and mental health [6]. Among university students, poor sleep quality and insomnia symptoms are common and appear to be linked to academic stress [7]. In the wake of the COVID-19 pandemic, several studies reported an increased prevalence of sleep disorders in this population [8, 9]. A recent German survey found that 48.7% of students reported poor subjective sleep quality [10]. 

Beyond their impact on mental health, sleep disturbances may also affect academic performance. For example, an association of poorer sleep quality prior to exams and worse first state medical exam results was demonstrated by a prior study [11]. However, evidence in this regard remains mixed: while some studies have similarly reported impaired academic performance among students with sleep disorders [12], others have paradoxically found higher academic performance among poor sleepers [13]. 

The individual chronotype is increasingly recognized as a critical factor for both academic and professional performance. The chronotype describes the individual circadian rhythm and associated preferred time window for wakefulness and activity. Individuals are generally classified as early (morning), late (evening), and intermediate types [14]. Late chronotypes are more likely to report insomnia symptoms across age groups and are at elevated risk for clinical insomnia [15, 16]. Late types furthermore show an increased risk of depression and anxiety disorders [17]. In academic settings, late chronotypes consistently perform worse than early or intermediate types, both at the school [18] and university level [19, 20]. 

In summary, sleep quality and its determinants are critically linked to the academic performance and mental health of medical students. However, the specific factors influencing sleep quality among medical students are not fully understood. This study therefore tested two hypotheses: (1) that medical students frequently experience reduced sleep quality and insomnia symptoms during exam periods, which may be accompanied by increased stress and psychiatric symptoms; and (2) that sleep quality is associated with both variable state markers (e.g., perceived stress and depressive symptoms) and (relatively) stable individual trait markers (e.g., chronotype and dysfunctional sleep beliefs). Importantly, dysfunctional sleep beliefs, although relatively stable as cognitive traits, are modifiable through targeted psychological interventions [21], and may therefore be a potential target for preventive approaches among students.

To address these questions, we conducted an anonymous cross-sectional survey among medical students during the exam period of the fourth year of medical school. We assessed prevalence and severity of insomnia symptoms alongside measures of stress, depressive symptoms, chronotype, dysfunctional sleep beliefs, and actual exam performance.

## Methods

### Study subjects and study design

This cross-sectional observational study was conducted at the Friedrich-Alexander-Universität Erlangen-Nürnberg to assess the prevalence of insomnia symptoms and associated factors among medical students. An anonymous survey was administered during the curricular exam period. Students aged ≥ 18 years who took the eighth-semester (fourth year of medical school) psychiatry examination in the 2024/25 winter term were invited to participate.

Participants were asked to complete the Insomnia Severity Index (ISI; prespecified primary outcome), selected items from the Pittsburgh Sleep Quality Index (PSQI), the reduced Morningness-Eveningness Questionnaire (rMEQ), the Dysfunctional Beliefs and Attitudes about Sleep Scale-16 (DBAS-16), the Patient Health Questionnaire-9 (PHQ-9), the Generalized Anxiety Disorder-2 (GAD-2), and a single-item visual analogue scale for perceived stress. Participation was voluntary, and informed consent was obtained at the start of the survey. As the study involved an anonymous questionnaire, a waiver was granted by the local ethics committee (Ethikkommission der Friedrich-Alexander-Universität Erlangen-Nürnberg, Krankenhausstraße 12, 91054 Erlangen; Reference No. 25-325-ANF), confirming that no formal vote was required. All procedures conformed to the principles of the Declaration of Helsinki.

### Questionnaires

All questionnaires used in this study were previously published, validated instruments; no new scales were developed for this study.

#### Insomnia Severity Index (ISI)

The Insomnia Severity Index (ISI) is a validated seven-item self-report instrument that assesses insomnia severity across multiple domains: difficulty initiating sleep, difficulty maintaining sleep, early morning awakenings, satisfaction with sleep patterns, interference with daily functioning, noticeability of impairment by others, and distress related to sleep disturbances [22]. Each item is scored on a 5-point Likert scale (0–4), with total scores ranging from 0 to 28; higher scores indicate greater insomnia severity [22]. Participants were dichotomized into insomnia and no-insomnia groups using a cut-off score of > 10, a threshold that optimally balances sensitivity and specificity in community-based populations [23, 24]. The ISI is a screening and severity instrument and does not replace a clinical interview, which is required to diagnose insomnia disorder. Accordingly, the terms “insomnia” and “insomnia group” in this manuscript refer to clinically relevant insomnia symptoms as identified by the ISI cut-off and not to a clinical diagnosis. For this study, the 4-week version of the validated German version of the ISI was used [25]. The ISI demonstrated good internal consistency in our sample (Cronbach’s α = 0.82).

#### Pittsburgh Sleep Quality Index (PSQI)

The Pittsburgh Sleep Quality Index (PSQI) is a validated self-report questionnaire that assesses sleep quality over the preceding month. The instrument comprises 19 items grouped into seven component scores: subjective sleep quality, sleep onset latency, sleep duration, sleep efficiency, sleep disturbances, use of sleep medication, and daytime dysfunction. Each component is scored from 0 to 3, yielding a global score ranging from 0 to 21, with higher scores indicating poorer sleep quality [26]. For this study, three sleep parameters derived from the PSQI were analyzed as continuous variables (rather than as 0–3 component scores): sleep onset latency (component 2; item 2, in minutes), sleep duration (component 3; item 4, reported hours of actual sleep, converted to minutes), and sleep efficiency (component 4). Sleep efficiency (%) was calculated as reported sleep duration divided by time in bed, multiplied by 100; time in bed was defined as the interval between habitual bedtime (item 1) and habitual rising time (item 3). In five instances where reported sleep duration exceeded the calculated time in bed, sleep efficiency was capped at 100%.

#### Reduced Morningness-Eveningness Questionnaire (rMEQ)

The reduced Morningness-Eveningness Questionnaire (rMEQ) is a validated five-item instrument derived from the original Morningness-Eveningness Questionnaire developed by Horne and Östberg [27]. The rMEQ assesses circadian chronotype by evaluating individual preferences for morning versus evening activity. Responses are scored on variable scales and summed to yield a total score ranging from 4 to 25, with higher scores indicating morning preference and lower scores indicating evening preference. Chronotype classification is based on the following thresholds: morning type (18–25 points), intermediate type (12–17 points), and evening type (4–11 points) [28, 29]. Internal consistency in our sample was acceptable (Cronbach’s α = 0.77).

#### Dysfunctional Beliefs and Attitudes about Sleep Scale-16 (DBAS-16)

The Dysfunctional Beliefs and Attitudes about Sleep Scale-16 is a 16-item self-report instrument that assesses maladaptive cognitions related to sleep and represents the short form of the Dysfunctional Beliefs and Attitudes about Sleep scale originally developed and validated by Morin and colleagues [21]. For this study, we used the validated German version [30]. Each item is rated on an 11-point scale (0–10), with higher scores reflecting stronger endorsement of dysfunctional sleep-related beliefs. The scale comprises four subscales, each reflecting a distinct domain of dysfunctional sleep cognitions. The Perceived Consequences subscale (items 5, 7, 9, 12, 16) includes questions such as “Without an adequate night’s sleep, I can hardly function the next day” and “I avoid or cancel obligations (social, family) after a poor night’s sleep”. The Worry/Helplessness subscale (items 3, 4, 8, 10, 11, 14) assesses beliefs like “I am concerned that chronic insomnia may have serious consequences on my physical health” and “When I sleep poorly on one night, I know it will disturb my sleep schedule for the whole week”. The Sleep Expectations subscale consists of item 1 (“I need 8 hours of sleep to feel refreshed and function well during the day”) and item 2 (“When I don’t get proper amount of sleep on a given night, I need to catch up on the next day by napping or on the next night by sleeping longer”). Lastly, the Medication Beliefs subscale (items 6, 13, 15) captures statements such as “I believe insomnia is essentially the result of a chemical imbalance” and “Medication is probably the only solution to sleeplessness”. The total DBAS-16 score is calculated as the mean of all 16 items, while subscale scores represent the mean of their respective items [21, 30]. In our sample, internal consistency was good (Cronbach’s α = 0.82).

#### Patient Health Questionnaire-9 (PHQ-9)

The Patient Health Questionnaire-9 (PHQ-9) is a widely used and validated nine-item self-report instrument that screens for and assesses the severity of depressive symptoms according to DSM-IV/V criteria for major depressive disorder [31]. For this study, we used the validated German version [32]. Each item is rated on a 4-point scale (0–3), with total scores ranging from 0 to 27 and higher scores indicating greater symptom severity. Depressive symptom severity is classified as follows: 0–4 points: no or minimal depressive symptoms; 5–9 points: mild depressive symptoms; 10–14 points: moderate depressive symptoms; 15–19 points: moderately severe depressive symptoms; 20–27 points: severe depressive symptoms [31, 32]. In this sample, internal consistency was good (Cronbach’s α = 0.87).

#### Generalized Anxiety Disorder-2 (GAD-2)

The Generalized Anxiety Disorder-2 (GAD-2) is a brief two-item screening instrument designed to identify probable cases of generalized anxiety disorder. The questionnaire assesses the frequency of anxiety and difficulty controlling worry over the preceding 4 weeks. Each item is rated on a 4-point scale (0–3), yielding total scores ranging from 0 to 6. A cut-off score of 3 or greater identifies a positive screen for clinically relevant anxiety symptoms (probable generalized anxiety disorder) [33]. Internal consistency in our sample was good (Cronbach’s α = 0.82).

#### Perceived stress

Perceived stress was assessed using a visual analogue scale (VAS) anchored from 0 (minimal stress) to 100 (maximal stress), with participants rating their subjective stress perception over the preceding 4 weeks. Higher scores indicated greater perceived stress. The VAS for perceived stress has demonstrated good discriminative sensitivity and construct validity in assessing perceived stress in previous studies [34]. 

### Academic performance

Academic performance was operationalized as the score achieved in the psychiatry examination of the eighth semester (fourth year of medical school), a curricular course examination that tested psychiatric knowledge and consisted of 30 multiple-choice items worth one point each, yielding a total score between 0 and 30 points; higher scores indicate better performance. Examination results were obtained from the examination office. Students obtained between 11 and 29 points. Overall difficulty of the questions was 0.79 and the mean item discrimination (corrected item-total correlation) was 0.14 according to the definition of Möltner et al. [35]. To enable anonymous linkage of survey and exam results, participants were asked to provide the unique identifier of the tablet used during the examination.

### Statistical analysis

Data were analyzed using IBM SPSS Statistics (version 30.0.0) and Microsoft Excel. All data entries were reviewed for completeness and consistency prior to analysis. Descriptive statistics included means and standard deviations for continuous variables and frequencies with percentages for categorical variables. For group comparisons between participants with and without insomnia, independent-samples t-tests were applied to continuous variables. Chi-square tests were used for categorical variables. Two multivariable linear regression models examined factors associated with insomnia severity (ISI total score). Missing data were handled using listwise deletion. The extent of missing data varied by variable, ranging from 1.6% (sleep parameters, chronotype) to 13.7% (PHQ-9, GAD-2, perceived stress) of the 124 participants (Table 2). Missing data resulted from incomplete or invalid questionnaire responses and, for examination performance, from incorrect entry of the tablet identifier, which prevented linkage of survey and examination data. The higher proportion of missing PHQ-9, GAD-2, and perceived stress data likely reflects their position toward the end of the questionnaire, consistent with increasing non-response over the course of the survey rather than symptom-related non-response. Because of listwise deletion, 27 participants (21.8%) were excluded from the primary regression model (*N* = 97) and 11 participants (8.9%) from the DBAS-16 subscale model (*N* = 113). The primary model included ten predictors (*N* = 97), while a secondary model (*N* = 113) included four DBAS-16 subscales. Chronotype was entered as a categorical variable with intermediate chronotype as the reference category, consistent with recent evidence demonstrating non-linear associations between chronotype and insomnia symptoms [36]. Model assumptions were verified through examination of residual plots, and multicollinearity was assessed using variance inflation factors. All reported P values were two-sided, with *P* < 0.05 considered statistically significant.

## Results

### Study population and baseline characteristics

**Fig. 1:**
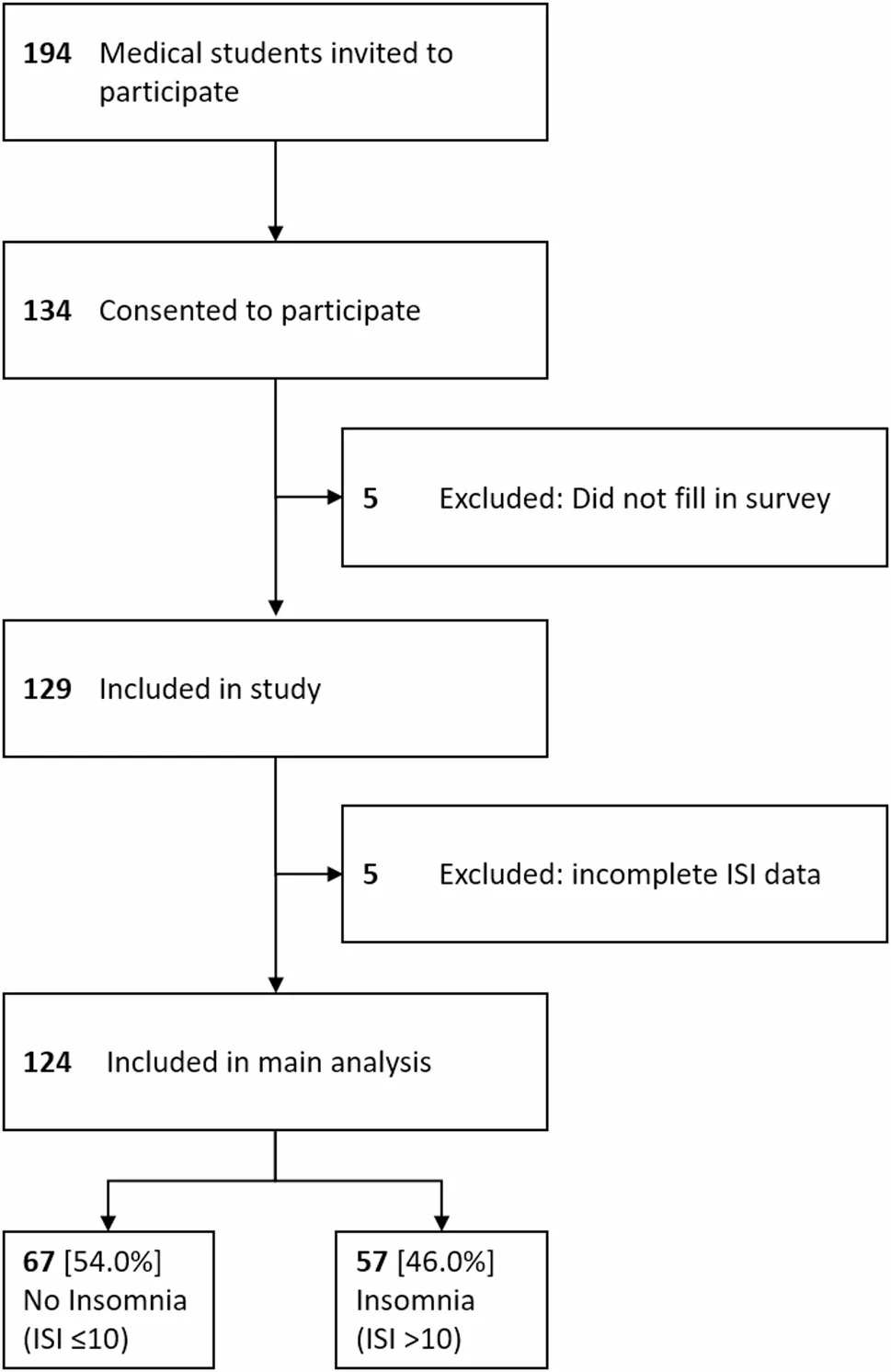
Participant flow chart. Of 134 enrolled participants, 10 were excluded due to incomplete data. Remaining participants were classified as without clinically relevant insomnia symptoms (n=67, 54.0%) or with clinically relevant insomnia symptoms (n=57, 46.0%) based on ISI score ≤10 or >10, respectively

A total of 194 medical students were invited to participate in the study. Of these, 134 consented to participate, yielding a response rate of 69.1% (Fig. 1).

Baseline demographics and clinical characteristics are presented in Table 1. The participants who completed the survey had a mean age of 25.2 [± 3.2] years. The majority of participants were female (97 [75.2%]), while 31 [24.0%] were male and 1 [0.8%] identified as other. After excluding 5 participants who did not complete the survey and a further 5 with incomplete ISI data, 124 participants were included in the main analysis.

**Table 1:** Baseline demographics and clinical characteristics

Variable	
Demographics	
Age, years	25.2 ± 3.2
Gender	
Female	97 (75.2)
Male	31 (24.0)
Other	1 (0.8)
Insomnia Severity (ISI)	
Total score	9.9 ± 4.9
No Insomnia (ISI ≤ 10)	67 (54.0)
Insomnia (ISI > 10)	57 (46.0)
Sleep Parameters (PSQI)	
Time in Bed, min	481.2 ± 68.3
Total Sleep Time, min	416.6 ± 71.0
Sleep Onset Latency, min	35.2 ± 31.9
Sleep Efficiency, %	86.6 ± 10.8
Chronotype (rMEQ)	
Total Score	13.1 ± 3.9
Intermediate Type	66 (54.1)
Evening Type	41 (33.6)
Morning Type	15 (12.3)
Dysfunctional Beliefs and Attitudes about Sleep (DBAS-16)	
Total Score	4.13 ± 1.4
Perceived Consequences	4.5 ± 1.6
Worry/Helplessness	4.1 ± 1.7
Sleep Expectations	6.9 ± 2.4
Medication Beliefs	1.7 ± 1.3
Depressive Symptoms (PHQ-9)	
Total Score	11.2 ± 5.6
Minimal (0–4)	11 (10.3)
Mild (5–9)	36 (33.6)
Moderate (10–14)	33 (30.8)
Moderately-Severe (15–19)	15 (14.0)
Severe (20–27)	12 (11.2)
Anxiety Symptoms (GAD-2)	
Total Score	2.4 ± 1.6
GAD-2 ≥ 3 (positive screen)	48 (44.9)
GAD-2 < 3	59 (55.1)
Perceived Stress, VAS (0-100)^a^	73.4 ± 14.6
Exam Performance, points	22.7 ± 2.1

Baseline demographics and clinical characteristics of the participants who completed the survey (*n* = 129). Continuous variables are presented as mean ± SD, categorical variables as number (percentage). Sample size varied by variable due to missing data: Age (*n* = 128), ISI (*n* = 124), sleep parameters (*n* = 122), chronotype (*n* = 122), DBAS-16 (*n* = 113), PHQ-9 (*n* = 107), GAD-2 (*n* = 107), perceived stress (*n* = 107), exam performance (*n* = 113)

*Abbreviations DBAS-16 * Dysfunctional Beliefs and Attitudes about Sleep Scale-16, *GAD-2 * Generalized Anxiety Disorder-2, *ISI * Insomnia Severity Index, *PHQ-9 * Patient Health Questionnaire-9, *PSQI * Pittsburgh Sleep Quality Index, *rMEQ * reduced Morningness-Eveningness Questionnaire, *SD * standard deviation, *VAS * visual analogue scale

^a^Perceived stress was assessed by using a visual analogue scale ranging from 0 (not stressed at all in the last 4 weeks) to 100 (extremely stressed)

Clinically relevant insomnia symptoms were highly prevalent: the mean ISI total score was 9.9 ± 4.9 points. Using the ISI cut-off score of > 10 to identify participants with clinically relevant insomnia symptoms [23, 37], 57 [46%] participants screened positive, whereas 67 [54%] participants scored ≤ 10. The mean time in bed was 481.2 ± 68.3 min, whereas the mean total sleep time was 416.6 ± 71.0 min. Participants reported a mean sleep onset latency of 35.2 ± 31.9 min, and the average sleep efficiency was 86.6% ± 10.8%.

The mean DBAS-16 score was 4.13 ± 1.4. Subscale analysis indicated that participants most strongly endorsed dysfunctional beliefs and attitudes regarding Sleep Expectations (6.9 ± 2.4), followed by Perceived Consequences (4.5 ± 1.6) and Worry/Helplessness (4.1 ± 1.7). Dysfunctional Medication Beliefs were less prevalent (1.7 ± 1.3).

Chronotype distribution was assessed using the reduced Morningness-Eveningness Questionnaire (rMEQ): 66 [54.1%] of the included participants were classified as intermediate type, 41 [33.6%] as evening type and 15 [12.3%] as morning type.

The mean PHQ-9 score was 11.2 ± 5.6 points. Regarding depression severity, 11 participants [10.3%] had no or minimal depressive symptoms, 36 [33.6%] mild, 33 [30.8%] moderate, 15 [14.0%] moderately severe, and 12 [11.2%] severe symptoms. The GAD-2 yielded a mean score of 2.4 ± 1.6, with 48 participants [44.9%] screening positive for anxiety symptoms. The perceived stress level, measured on a visual analogue scale from 0 to 100, showed a mean score of 73.4 ± 14.6.

### Comparison of participants with and without clinically relevant insomnia symptoms

Participants were stratified into an insomnia group (ISI total score > 10; *N* = 57) and a non‑insomnia group (ISI total score ≤ 10; *N* = 67) (Table 2). Age and gender did not differ significantly between the groups.

**Table 2:** Comparison of participants with and without clinically relevant insomnia symptoms

Variable	Participants without Insomnia Symptoms *n* = 67	Participants with Insomnia Symptoms *n* = 57	*P*
Demographics			
Age, years	25.5 ± 3.6	25.0 ± 2.7	0.39
Gender			
Female	49 (73.1)	45 (78.9)	0.40
Male	18 (26.9)	12 (21.1)	0.40
Sleep Parameters (PSQI)			
Time in Bed, min	485.5 ± 61.1	476.3 ± 76.1	0.47
Total Sleep Time, min	438.2 ± 58.3	391.0 ± 76.5	< 0.001
Sleep Onset Latency, min	21.6 ± 18.0	51.1 ± 37.1	< 0.001
Sleep Efficiency, %	90.1 ± 7.2	82.2 ± 12.2	< 0.001
Chronotype (rMEQ)			
Total Score	13.6 ± 3.5	12.6 ± 4.3	0.20
Intermediate Type	43 (65.2)	23 (41.1)	0.01
Evening Type	15 (22.7)	26 (46.4)	0.009
Morning Type	8 (12.1)	7 (12.5)	0.93
Dysfunctional Beliefs and Attitudes about Sleep (DBAS-16)			
Total Score	3.5 ± 1.3	4.8 ± 1.1	< 0.001
Perceived Consequences	3.8 ± 1.6	5.3 ± 1.4	< 0.001
Worry/Helplessness	3.4 ± 1.7	5.0 ± 1.3	< 0.001
Sleep Expectations	6.3 ± 2.7	7.0 ± 2.2	0.1
Medication Beliefs	1.3 ± 1.1	2.2 ± 1.4	< 0.001
Depressive Symptoms (PHQ-9 Total Score)	8.6 ± 4.6	14.1 ± 5.4	< 0.001
Anxiety Symptoms (GAD-2 Total Score)	1.9 ± 1.4	3.0 ± 1.7	< 0.001
Perceived Stress, VAS (0-100)^a^	69.3 ± 15.5	78.0 ± 11.9	0.002
Exam Performance	22.9 ± 1.9	22.6 ± 2.3	0.41

Continuous variables are presented as mean ± SD, categorical variables as number (percentage). Sample size varied by variable due to missing data: ISI (*n* = 124), sleep parameters (*n* = 122), chronotype (*n* = 122), DBAS-16 (*n* = 113), PHQ-9 (*n* = 107), GAD-2 (*n* = 107), perceived stress (*n* = 107), exam performance (*n* = 111). Group comparisons for categorical variables were conducted using Pearson’s Chi-Square Test, whereas independent samples t-tests were used for continuous variables. No Fisher’s Exact Test was necessary, as all expected cell counts were ≥ 5. A *p*-value < 0.05 was considered statistically significant

*Abbreviations  DBAS-16 * Dysfunctional Beliefs and Attitudes about Sleep Scale-16, *GAD-2 * Generalized Anxiety Disorder-2, *ISI * Insomnia Severity Index, *PHQ-9 * Patient Health Questionnaire-9, *PSQI* Pittsburgh Sleep Quality Index, *rMEQ * reduced Morningness-Eveningness Questionnaire, *SD * standard deviation, *VAS * visual analogue scale

^a^Perceived stress was assessed by using a visual analogue scale ranging from 0 (not stressed at all in the last 4 weeks) to 100 (extremely stressed)

Participants with clinically relevant insomnia symptoms reported significantly shorter total sleep time (391.0 ± 76.5 min vs. 438.2 ± 58.3 min, *P* < 0.001), longer sleep onset latency (51.1 ± 37.1 min vs. 21.6 ± 18.0 min, *P* < 0.001), and lower sleep efficiency (82.2 ± 12.2% vs. 90.1 ± 7.2%, *P* < 0.001) compared to participants without clinically relevant insomnia symptoms.

Dysfunctional beliefs and attitudes about sleep were significantly more prevalent in participants with clinically relevant insomnia symptoms, as reflected by higher DBAS-16 mean scores (4.8 ± 1.1 vs. 3.5 ± 1.3, *P* < 0.001) in the insomnia group. Subscale analysis revealed significantly higher mean scores in the insomnia group for Perceived Consequences (5.3 ± 1.4 vs. 3.8 ± 1.6, *P* < 0.001), Worry/Helplessness (5.0 ± 1.3 vs. 3.4 ± 1.7, *P* < 0.001) and Medication Beliefs (2.2 ± 1.4 vs. 1.3 ± 1.1, *P* < 0.001). Sleep Expectations showed no significant differences (7.0 ± 2.2 vs. 6.3 ± 2.7, *P* = 0.1).

Chronotype classification (rMEQ) revealed that evening types were significantly more frequent in the insomnia group (46.4% vs. 22.7%, *P* = 0.009), while intermediate types were predominant in the non-insomnia group (65.2% vs. 41.1%, *P* = 0.01).

Participants with clinically relevant insomnia symptoms also reported higher levels of stress during the last 4 weeks (visual analogue scale of perceived stress: 78.0 ± 11.9 vs. 69.3 ± 15.5, *P* = 0.002), greater depressive symptoms (mean PHQ-9 score: 14.1 ± 5.4 vs. 8.6 ± 4.6, *P* < 0.001), and higher anxiety symptoms (mean GAD-2 score: 3.0 ± 1.7 vs. 1.9 ± 1.4, *P* < 0.001). Exam performance did not significantly differ between groups.

### Multivariable linear regression analysis of factors associated with insomnia severity

A multivariable linear regression was used to identify variables associated with insomnia severity, as measured by the ISI total score (*N* = 97; Table 3). All variables were entered simultaneously using the inclusion method. The overall model was statistically significant, F(10, 86) = 11.51, *P* < 0.001 and explained a substantial proportion of variance in ISI scores (R² = 0.572; adjusted R² = 0.523), delivering a strong model fit according to Cohen’s conventions [38]. Examination of residuals revealed no evidence of autocorrelation (Durbin-Watson statistic = 1.906), no severe deviations from normality or homoscedasticity, and no influential outliers requiring removal. Regarding multicollinearity, all Variance Inflation Factors (VIF) were below the critical threshold of 10 (range: 1.04–2.57), consistent with zero-order correlation results (highest *r* = 0.68 between PHQ-9 and GAD-2). Four variables were independently associated with insomnia severity. Dysfunctional sleep beliefs (DBAS-16, β = 0.28, *P* < 0.001) and depressive symptoms (PHQ-9, β = 0.30, *P* = 0.01) were associated with increased severity, whereas higher sleep efficiency (β = −0.30, *P* < 0.001) and better examination performance (β = −0.15, *P* = 0.037) were associated with reduced severity. Age, gender, GAD-2 total score, chronotype and perceived stress were not significantly associated with insomnia severity when controlling for the other variables. Results remained stable in a sensitivity analysis excluding sleep-related items from the PHQ-9 (Supplementary Table 1). The associations of PHQ-9 (*P* = 0.045) and examination performance (*P* = 0.049) were, however, only of borderline significance in this analysis.

**Table 3:** Multivariable linear regression analysis of factors associated with insomnia severity

Variable	B	Std. Error B	β (Beta)	t	*P*
Age	-0.17	0.12	-0.11	-1.41	0.16
Female Gender	0.73	0.82	0.07	0.90	0.37
Exam Performance	-0.35	0.16	-0.15	-2.12	0.037
Sleep Efficiency (%)	-0.13	0.04	-0.30	-3.82	< 0.001
Chronotype: Morning Type	0.74	1.20	0.05	0.62	0.54
Chronotype: Evening Type	0.80	0.77	0.08	1.05	0.30
Dysfunctional Beliefs (DBAS-16)	1.07	0.31	0.28	3.42	< 0.001
Depressive Symptoms (PHQ-9)	0.26	0.10	0.30	2.61	0.01
Anxiety Symptoms (GAD-2)	0.18	0.33	0.06	0.55	0.58
Perceived Stress	0.04	0.03	0.12	1.33	0.19
*Constant*	*21.91*	*6.42*	*-*	*3.41*	*< 0.001*

Insomnia Severity Index (ISI) total scores regressed on psychological, behavioral, and sleep-related variables (*N* = 97; R² = 0.572; adjusted R² = 0.523; F(10, 86) = 11.51; *P* < 0.001). Missing data were handled using listwise deletion

### Multivariable linear regression analysis of DBAS-16 subscales associated with insomnia severity

In an additional analysis using identical methodology, a multivariable linear regression model was conducted to examine the independent contribution of DBAS-16 subscales to insomnia severity, as measured by the ISI total score (*N* = 113; Table 4). The overall model was statistically significant, F(4, 108) = 13.43, *P* < 0.001, and explained 33.2% of the variance in ISI scores (R² = 0.332; adjusted R² = 0.307), equivalent to a large effect size according to Cohen’s conventions. Model diagnostics confirmed the validity of regression assumptions: no evidence of autocorrelation (Durbin-Watson statistic = 1.914), no influential outliers, and Variance Inflation Factors within acceptable ranges (1.29–1.87), indicating negligible multicollinearity. Residual plots revealed no severe deviations from normality or homoscedasticity. The “Worry/Helplessness” subscale showed the strongest association (β = 0.35, *P* = 0.001), followed by the “Perceived Consequences” subscale (β = 0.22, *P* = 0.04). In contrast, “Sleep Expectations” (β = -0.10, *P* = 0.29) and “Medication Beliefs” (β = 0.17, *P* = 0.06) were not significant when controlling for the other subscales.

**Table 4:** Linear regression model of insomnia severity on dysfunctional beliefs and attitudes about sleep

Variable	B	Std. Error B	β (Beta)	t	*P*
Perceived Consequences	0.66	0.32	0.22	2.04	0.04
Worry/Helplessness	1.04	0.31	0.35	3.34	0.001
Sleep Expectations	-0.19	0.18	-0.10	-1.07	0.29
Medication Beliefs	0.63	0.33	0.17	1.89	0.06
*Constant*	*2.97*	*1.33*	*-*	*2.24*	*0.03*

Insomnia Severity Index (ISI) total scores regressed on DBAS-16 subscales (*N* = 113; R² = 0.332; adjusted R² = 0.307; F(4, 108) = 13.43; *P* < 0.001). Missing data were handled using listwise deletion

## Discussion

This study aimed to investigate the factors associated with sleep quality among medical students during a high-stress examination period, with a particular focus on the relative associations of state factors (e.g., perceived stress, depressive symptoms) and more stable trait markers (chronotype, dysfunctional sleep beliefs) with insomnia severity.

During the curricular examination period, medical students reported reduced sleep quality. Adjusted analyses using multivariable linear regression identified depressive symptoms and dysfunctional beliefs about sleep as key independent correlates of higher insomnia severity, with sleep efficiency and exam performance inversely related.

Insomnia symptoms were very prevalent during the exam period: 46% of medical students assessed met the ISI threshold for clinically relevant insomnia symptoms, which markedly exceeds estimates from general population cohorts [39, 40] and aligns with recent data in similar student populations [10]. Sleep parameters collected across the entire cohort indicated a mild but relevant reduction in overall sleep quality. The mean total sleep time was marginally below the recommended minimum of seven hours [41], and given that sleep duration is often overestimated in self-report [42], the true extent of sleep deficiency in this cohort may be underestimated. Mean sleep efficiency (86.6%) was at the lower bound of population values [43, 44]. Mean sleep onset latency (35.2 min) exceeded the conventional threshold of 30 min that defines clinically significant difficulties in falling asleep [44–46]. 

When comparing students with clinically relevant insomnia symptoms (ISI > 10) to those without, sleep continuity parameters showed significant impairment. Specifically, students with insomnia had reduced total sleep time, prolonged sleep onset latency, and decreased sleep efficiency, all deviating from normative values [44, 47]. Consistently, sleep efficiency was independently associated with insomnia severity in the multivariable model, aligning with literature that identifies reduced sleep efficiency as a key feature of insomnia [48, 49]. Collectively, these findings indicate substantial impairment in both perceived and continuity‑based indices of sleep during the exam period.

Because data were collected during an exam period characterized by heightened psychosocial stress, we considered whether situational (‘state’) factors versus more stable (‘trait’) characteristics were differentially associated with the observed sleep impairment. As state markers linked to the examination context, we evaluated perceived stress, depressive and anxiety symptoms, and examination performance. As trait markers less susceptible to short-term stress, we assessed chronotype and dysfunctional beliefs about sleep. This distinction is conceptual: because all variables were assessed at a single time point, state and trait components could not be separated empirically.

Within the state domain, depressive symptoms emerged as a consistent correlate of insomnia severity in both unadjusted and adjusted analyses. Depressive symptoms were elevated among students with insomnia compared with those without, and higher PHQ-9 scores were independently associated with greater insomnia severity in the adjusted multivariable regression. The depressive burden in our cohort exceeded population norms [50] and, even outside the insomnia group, matched or surpassed levels reported in German students during the COVID-19 pandemic [51]. The association between depression and insomnia severity remained robust when the two sleep-related PHQ-9 items were excluded, and aligns with prior work linking insomnia and depression [6, 52–54]. Taken together, these patterns highlight a significant overlap between depressive symptoms and insomnia severity during the examination period, consistent with previous reports of impaired sleep during exam periods [55]. 

Although perceived stress and anxiety were elevated among students with insomnia, neither remained independently associated with insomnia severity after adjustment. This pattern suggests that their associations were largely accounted for by variance shared with depressive symptoms rather than a distinct effect. Supporting this interpretation, anxiety and perceived stress correlated with depression in our cohort (*r* ≈ 0.68 for GAD-2/PHQ-9; *r* ≈ 0.49 for perceived stress/PHQ-9) and in prior studies [56–58]. These observations are consistent with the high comorbidity of depression and anxiety and with models positing a shared negative affect factor across the two disorders [59, 60]. 

Regarding academic performance, mean examination scores did not differ between students with and without insomnia. Nevertheless, in the adjusted multivariable regression with insomnia severity as the outcome, examination scores were inversely associated with insomnia severity. This pattern is compatible with a modest association that may be obscured in dichotomous group comparisons by range restriction and exam characteristics. The present outcome, a 30-item, domain-limited course test with inherently narrow score dispersion, is unlikely to capture subtle decrements in academic functioning. Accordingly, it is not directly comparable to the comprehensive, high-stakes first state medical examination studied by Ahrberg et al. [11], where poorer sleep and higher stress before the exam were associated with lower scores. Experimental and observational data in student populations similarly connect sleep quality with cognitive performance [61, 62]. 

In the trait domain, chronotype showed the expected unadjusted pattern: evening types were overrepresented among students with insomnia, whereas intermediate types were more frequent among those without insomnia, consistent with prior reports linking eveningness to insomnia risk [15, 16]. However, in the adjusted multivariable models neither chronotype category nor the rMEQ total score remained independently associated with insomnia severity, suggesting that the apparent chronotype association may be attenuated after accounting for depressive symptoms, dysfunctional sleep beliefs, and sleep-continuity parameters.

In contrast to chronotype, dysfunctional beliefs and attitudes about sleep were robustly associated with insomnia. Students with insomnia scored significantly higher on the DBAS-16 total and on several subscales (Perceived Consequences, Worry/Helplessness, Medication Beliefs). In the adjusted multivariable regression, the DBAS-16 total score remained an independent correlate of greater insomnia severity. These findings are consistent with earlier studies linking dysfunctional attitudes and beliefs to insomnia and suggesting that they may contribute to its persistence [21, 63, 64]. 

When the four DBAS-16 subscales were examined independently, Worry/Helplessness and Perceived Consequences emerged as statistically significant correlates of insomnia severity, whereas Sleep Expectations and Medication Beliefs did not reach statistical significance. Notably, the subscale model alone accounted for a substantial share of variance, suggesting that worry about sleep control and perceived negative consequences of insomnia may be the aspects of dysfunctional sleep beliefs most closely associated with insomnia severity in this population. Given the cross-sectional design, however, these findings demonstrate associations only; they do not allow conclusions about underlying mechanisms or the direction of the relationship, and insomnia symptoms may themselves foster dysfunctional beliefs. The prominence of Worry/Helplessness and Perceived Consequences is compatible with cognitive models of insomnia, which posit that worry about sleep and its perceived consequences trigger arousal and selective attention toward sleep-related threat cues, thereby creating a self-perpetuating cycle of sleep disturbance [65]. 

These findings are noteworthy because dysfunctional sleep beliefs were prevalent across the entire cohort despite formal medical training, which was expected to include education about sleep physiology. This observation suggests that deficient sleep health literacy may be a systemic issue within medical education, not merely an individual health problem. Clinician attitudes and beliefs substantially influence clinical decision-making and patient management [66, 67]. Consequently, medical students with dysfunctional sleep cognitions may perpetuate suboptimal insomnia care in their future practice. Accordingly, clinical practice reveals substantial deficits in insomnia management, including overreliance on hypnotic medication [68, 69], despite clear guideline support for cognitive behavioral therapy for insomnia (CBT-I) as first-line treatment [70]. 

The prevalence of dysfunctional sleep beliefs among medical students suggests that educational approaches on sleep health, for example brief psychoeducational modules addressing common sleep-related misconceptions or CBT-I–informed curricula, might be worth exploring. If effective, such interventions might yield dual benefits: They could improve the trainees’ own sleep quality while enhancing their preparedness to implement evidence-based, non-pharmacologic approaches to insomnia in future clinical practice. Whether this is the case remains to be tested in intervention studies.

### Strengths and limitations

This study’s strengths include a comprehensive real-world design with data collection during a clinically relevant period of high psychosocial stress, multidomain assessment using validated instruments across sleep and mental health domains, the inclusion of objective academic performance metrics, and a multivariable analytical approach that simultaneously examined multiple predictors while controlling for confounding. However, several limitations warrant consideration. The cross-sectional design precludes causal inference regarding the directionality among sleep, mood, and academic outcomes; the reported associations do not allow conclusions about mechanisms, and the relationships may be bidirectional. Reliance on self-reported sleep parameters, rather than objective measures such as polysomnography or actigraphy, may introduce recall bias. As all variables except academic performance were assessed by self-report at a single time point, shared method variance may have inflated the observed associations; the anonymous survey and the use of different validated instruments are likely to limit this bias. The ISI, PHQ-9, and GAD-2 are validated screening and severity instruments that do not replace a clinical interview; our findings therefore refer to symptom levels above screening cut-offs and not to diagnosed disorders, and other sleep disorders that can raise ISI scores (e.g., sleep-disordered breathing, circadian rhythm sleep–wake disorders) [71] were not assessed. The single-center, academically homogeneous sample limits generalizability beyond this specific population and examination period. Moreover, while the study achieved an acceptable response rate of 69%, selection bias cannot be excluded, and participants may differ systematically from non-respondents regarding their sleep and stress profiles. Technical limitations include range restriction introduced by the 30-item examination format, which may have attenuated observed associations with academic performance. Finally, listwise deletion (mainly due to incomplete questionnaires and unmatched examination data) reduced the regression models to *N* = 97 and *N* = 113; smaller associations, such as that with academic performance, should therefore be interpreted with caution.

### Future directions

Prospective longitudinal designs are needed to establish directionality and examine temporal relationships among insomnia severity, depressive symptoms, and academic performance. Randomized controlled trials of brief CBT-I or psychoeducational interventions targeting dysfunctional sleep beliefs would clarify whether addressing these cognitions yields benefits for both student well-being and clinical competence in insomnia management.

## Conclusion

Nearly half of fourth-year medical students surveyed screened positive for clinically relevant insomnia symptoms during an examination period. Depressive symptoms and dysfunctional sleep beliefs were independently associated with greater insomnia severity, whereas sleep efficiency and examination performance were inversely related. These findings suggest that sleep and mental health merit attention in medical education. Given the prominence of dysfunctional sleep beliefs, brief curricula in sleep medicine and non-pharmacologic insomnia care could be evaluated in future studies for their potential to benefit students’ own sleep and to prepare them for the management of insomnia in clinical practice.

## Supplementary Information

Supplementary Material 1.

## Data Availability

Datasets can be obtained from the corresponding author upon reasonable request.

## Abbreviations

CBT-I: Cognitive Behavioral Therapy for Insomnia
COVID-19: Coronavirus Disease 2019
DBAS-16: Dysfunctional Beliefs and Attitudes about Sleep Scale-16
DSM-IV/V: Diagnostic and Statistical Manual of Mental Disorders, Fourth/Fifth Edition
GAD-2: Generalized Anxiety Disorder-2
ISI: Insomnia Severity Index
PHQ-9: Patient Health Questionnaire-9
PSQI: Pittsburgh Sleep Quality Index
rMEQ: Reduced Morningness–Eveningness Questionnaire
SD: Standard Deviation
VAS: Visual Analogue Scale
VIF: Variance Inflation Factor
