# Supplementary material for: Insomnia in medical students during an examination period: associations with depression, dysfunctional sleep beliefs, and academic performance
Source: BMC Med Educ. 2026 Oct 12;26:1642. doi: 10.1186/s12909-026-10565-4 (PMC13660075; doi:10.1186/s12909-026-10565-4)
Supplement: Supplementary file 1 [file 12909_2026_10565_MOESM1_ESM.docx]

Supplementary Material

# 1 Supplementary Table 1. Linear regression analysis predicting ISI scores with modified PHQ-9 variable

| **Predictor Variable** | **B** | **Std. Error B** | **β (Beta)** | **t** | **P** |
| --- | --- | --- | --- | --- | --- |
| Age | -0.19 | 0.13 | -0.12 | -1.52 | 0.13 |
| Female Gender | 0.98 | 0.82 | 0.09 | 1.19 | 0.24 |
| Exam Performance | -0.33 | 0.17 | -0.15 | -1.99 | **0.049** |
| Sleep Efficiency (%) | -0.14 | 0.04 | -0.32 | -4.13 | **<0.001** |
| Chronotype: Morning Type | 0.73 | 1.22 | 0.05 | 0.59 | 0.55 |
| Chronotype: Evening Type | 0.97 | 0.77 | 0.1 | 1.26 | 0.21 |
| Dysfunctional Beliefs (DBAS-16) | 1.15 | 0.32 | 0.30 | 3.66 | **<0.001** |
| Depressive Symptoms (PHQ-9) | 0.25 | 0.12 | 0.23 | 2.03 | **0.045** |
| Anxiety Symptoms (GAD-2) | 0.23 | 0.34 | 0.08 | 0.68 | 0.50 |
| Perceived Stress | 0.05 | 0.03 | 0.14 | 1.53 | 0.13 |
| *Constant* | *22.62* | *6.52* | *-* | *3.47* | *<0.001* |

A multiple linear regression was conducted to examine whether the relationships between insomnia severity and relevant predictors remained stable when using a modified PHQ-9 score, excluding the sleep-related items 3 and 4. Examination of residuals revealed no evidence of autocorrelation (Durbin-Watson = 1.893). Residual plots indicated no severe deviations from normality or homoscedasticity. All Variance Inflation Factors (VIFs) were well below the critical threshold of 10 (highest VIF = 2.46, PHQ-9 *modified*), suggesting no problematic multicollinearity.

The model explained 55.9% of the variance in ISI scores (R²= 0.559, adjusted R²= 0.508) and was statistically significant, F(10, 86) = 10.92, P < 0.001.

Comparing to the main analysis, the same four variables emerged as statistically significant predictors, DBAS-16 total score, (modified) PHQ-9 total score, sleep efficiency and exam total score. Higher levels of dysfunctional beliefs about sleep and depressive symptoms (excluding sleep-related items) were still associated with greater insomnia severity, while higher sleep efficiency and better exam performance still predicted lower ISI scores significantly. Age, gender, chronotype, GAD-2 total score and perceived stress were not significant predictors when controlling for the other variables.
